# Formulation and Optimization of Sodium Alginate Polymer Film as a Buccal Mucoadhesive Drug Delivery System Containing Cetirizine Dihydrochloride

**DOI:** 10.3390/pharmaceutics13050619

**Published:** 2021-04-26

**Authors:** Krisztián Pamlényi, Katalin Kristó, Orsolya Jójárt-Laczkovich, Géza Regdon

**Affiliations:** Institute of Pharmaceutical Technology and Regulatory Affairs, University of Szeged, Eötvös u. 6., H-6720 Szeged, Hungary; pamlenyi.krisztian@szte.hu (K.P.); kristo.katalin@szte.hu (K.K.); jojartne.laczkovich.orsolya@szte.hu (O.J.-L.)

**Keywords:** buccal, mucoadhesive, drug delivery system, polymer film, alginate, cetirizine, FTIR, RAMAN mapping, statistical analysis

## Abstract

Currently, pharmaceutical companies are working on innovative methods, processes and products. Oral mucoadhesive systems, such as tablets, gels, and polymer films, are among these possible products. Oral mucoadhesive systems possess many advantages, including the possibility to be applied in swallowing problems. The present study focused on formulating buccal mucoadhesive polymer films and investigating the physical and physical–chemical properties of films. Sodium alginate (SA) and hydroxypropyl methylcellulose (HPMC) were used as film-forming agents, glycerol (GLY) was added as a plasticizer, and cetirizine dihydrochloride (CTZ) was used as an active pharmaceutical ingredient (API). The polymer films were prepared at room temperature with the solvent casting method by mixed two-level and three-level factorial designs. The thickness, tensile strength (hardness), mucoadhesivity, surface free energy (SFE), FTIR, and Raman spectra, as well as the dissolution of the prepared films, were investigated. The investigations showed that GLY can reduce the mucoadhesivity of films, and CTZ can increase the tensile strength of films. The distribution of CTZ proved to be homogeneous in the films. The API could dissolve completely from all the films. We can conclude that polymer films with 1% and 3% GLY concentrations are appropriate to be formulated for application on the buccal mucosa as a drug delivery system.

## 1. Introduction

Nowadays, the buccal drug delivery system is becoming an important alternative route in drug administration. This possibility of drug application has been untapped and poorly investigated. A buccal drug delivery system has some bioadhesive solid dosage forms, such as bioadhesive tablets, gels, patches, and mucosal bioadhesive films [1,2,3]. The patients have to place the bioadhesive system onto the buccal mucosa of the mouth. According to Macedo and Patel, this drug delivery system possesses great advantages. A lower dose of API can be applied compared with other delivery systems. Since the first-pass effect of the liver is avoided, the API can enter the systemic circulation after absorption from the buccal mucosa. Secondly, it can be used in the case of swallowing problems, such as Parkinson’s disease, stroke, multiple sclerosis, esophagitis, and allergies [4,5]. In geriatrics and pediatrics, it can play a huge role due to its easy and unnoticed application, so patients prefer this dosage form to per os tablets [6,7]. It is a useful form of drug administration from the chemical perspective, because the API and gastric acid do not contact each other, so the stomach can be protected from the API and vice versa [8,9]. However, this dosage form also has difficulties. In the polymer film, a dose of less than 20 mg of the API can be used successfully [10] due to the fact that the surface of the buccal mucosa is small, so a small dose of API can be absorbed from the small absorption surface. Buccal polymer films might be preferred to buccal tablets. They are more flexible and comfortable as they can be washed from the buccal mucosa by the saliva and, thus, be removed easily [5]. Oral and buccal controlled drug delivery systems are used widely with hydrophilic polymers [11,12]. Oral buccal polymer films must have at least medium mucoadhesive strength; therefore, one or more polymer film-forming excipients, such as sodium alginate (SA) and hydroxypropyl methylcellulose (HPMC), can be chosen [8].

SA is a natural water-soluble, nontoxic, biodegradable anionic polymer extracted from brownseeds (Phaeophyceae), *Macrocystis Pyriferal* and *Ascophyllum Nodosum* [13,14]. It is a member of the polysaccharides (linear) chemical family. It is built by two types of monomers, *β*-D mannuronic acid (1–4) (M) and *α*-L guluronic acid (1–6) (G). G and M can be arranged as homopolymer G blocks and M blocks or as heteropolymer mixed blocks (MG) [14]. SA has hydroxyl and carboxyl groups, which can bind to the mucin of the buccal mucosa [15]. The G blocks of SA can form a gel with divalent ions. SA is used in the pharmaceutical and the food and beverage industry. In the pharmaceutical industry, it is often used as a coating component, gel and emulsion stabilizing, and polymer film-forming excipient. The listed properties make SA suitable to be applied in buccal mucoadhesive films [14,15,16,17].

In our previous work, the film-forming properties of HPMC were studied [18]. HPMC is a popular, water-soluble, semisynthetic polymer. It is a white, solid, nontoxic polysaccharide (cellulose ether) molecule. In the literature, many researchers have reported that HPMC has good mucoadhesive properties [19,20,21,22]. Due to its large number of methoxy, carboxyl, and hydroxyl groups, it can create stable H-bondings with the mucin of the buccal mucosa. Kumria et al. concluded that the larger HPMC content can raise the mucoadhesion time and mucoadhesion force of films [23]. According to Peh and Wong, HPMC films swelled to a greater extent in simulated saliva solution [24]. This fact proves the great mucoadhesivity of HPMC. HPMC is a polymer with high hydrophilicity for an oral-controlled drug delivery system [20,21,25]. It is used as a film-forming agent, thickener agent, coating agent, and alternative to gelatin, and it is a polymer matrix system in tablets, capsules, gels, and eye drops. In addition, it is applied in agriculture, food, and cosmetics [26].

Glycerol (GLY) is an often-used liquid, which has colorless, viscous, nontoxic (dose-dependent), and sweet-tasting properties. GLY belongs to the family of polysaccharides. In polymer chemistry, GLY is used as a plasticizer, because it can make the film soft, flexible, and elastic [27,28,29,30].

Cetirizinedihydrochloride (CTZ), which is a second-generation, nonsedative drug, is a generally used antihistamine [31,32,33]. It is a member of BCS I classification. CTZ has three ionizable groups, a strong basic amino group, a strong carboxylic acid group, and a weak basic amino group, and it is a zwitterion structure between pH 3.0 and 8.0 [34]. CTZ has two remarkable forms (conformers), a folded and an extended one. The folded conformer has notable lipophobicity, low polarity, and a very low energy form due to the resulting intramolecular ionic bond between the positive and the negative charges. However, the extended form is characterized by high polarity, good lipophilicity, and it can form intramolecular hydrogen bindings, Van der Waals forces, and ionic bonds [35]. CTZ is applied in the case of allergic reactions (urticaria or rhinitis) and asthma bronchial. In addition, it is used intravenously in emergencies (anaphylaxia) by paramedics after the administration of adrenalin and intravenous steroids [36,37].

Our work was focused on preparing buccal mucoadhesive polymer films, which contained SA and CTZ. Many research groups used SA as a polymer film-forming agent to formulate buccal films, but it is not a commonly applied polymer material, unlike cellulose derivatives [15,16,17]. CTZ is an often-used API. It is available as oral tablets and in liquid forms in the case of an allergic reaction. Patients are able to use it easily, and it can have a fast effect without paramedic assistance; therefore, it is worth formulating and investigating in buccal films. Phalguna et al. and Baniya et al. formulated similar buccal films, but the mechanical properties of the films were not investigated as thoroughly as this dosage form requires. On the other hand, they did not examine the chemical interactions between the excipients in the film at all, although it is important to investigate them, because interactions may arise between the excipients of the films or the structure of the API may change. The mucoadhesivity of the films was not studied either, despite the fact that it is the criterion of the application. This phenomenon can fundamentally influence the mechanical, chemical, and drug release properties of films [38,39].

Our main aim was to prepare films with different compositions and evaluate the physicochemical investigation of buccal mucoadhesive films containing CTZ and SA by mixed two-level and three-level factorial designs. The interactions between the excipients and the API of the films, which can influence the mechanical and drug release properties of films, were investigated with several methods, and our further aim was to investigate the effect of the GLY content on the API distribution. Statistical analysis (factorial design) was also used to evaluate the data of the films. With this analysis, we are able to predict the properties of certain compositions without measurement, so the efficiency can increase. Moreover, we also aimed to investigate, by using several methods, the possible interactions between the excipients and the API of the films, which can influence the mechanical and drug release properties of the films. Based on these results and our opinion, depending on the composition, CTZ-containing buccal films can be applied in the case of allergic reactions. On the one hand, our goal was to find the optimal structure of the mucoadhesive film; on the other hand, CTZ-containing buccal films, depending on the composition, are well-applicable in the case of allergic reactions.

Therefore, our goal was to prepare films that can have their effect under 2 to 3 min. In the case of our present formulations, the prepared films can be used for the treatment of rhinitis, conjunctivitis, or other general symptoms of allergies.

## 2. Materials and Methods

### 2.1. Materials

SA (AppliChem GmbH, Darmstadt, Germany) (10,000–600,000 g/mol) and HPMC (Pharmacoat^®^ 603, Shin Etsu Chemical Co., Ltd., Tokyo, Japan) were used as a film-forming agent in the polymer film. GLY 85% (*w/w* %) was added to the film as a plasticizer [Ph. Eur. 8.], CTZ [Ph. Eur. 8.], which was a gift from ExtractumPharma Pharmaceutical Manufacturing, Marketing and Consulting Inc, Kunfehértó, Hungary was the API in the polymer film. Mucin (Carl Roth GmbH + Co. KG, Karlsruhe, Germany) (10 *w/w* %) dispersion was used in the in vitro mucoadhesion test. Diiodomethane was used in the surface free energy measurement.

### 2.2. Preparation of Films

The films were prepared at room temperature with the solvent casting method. As the first step of preparation, SA (1, 1.33, 1.5, and 2 *w/w* %) was solved in distilled water and mixed (900× *g* rpm) at room temperature. The solution was heated to 70 °C and mixed (900× *g* rpm), and CTZ was solved in the warm solution (0.5523 g/100 g solution) for 5 h. As the third step, HPMC (0.66:1:1.5 *w/w* %) was added to the solution with mixing without heating. In the fourth step, GLY was added to the solution the following day. Mixing was decreased to 100× *g* rpm for 3 h to make the air bubbles disappear from the solution. The solution was cast on a circular Teflon surface (diameter: 7.6 cm, area: 45.34 cm^2^) in rubber rings, with 10 g of solution/ring; then, it was dried at room temperature (24.4 ± 0.5 °C). The dried polymer films were removed from the surface and the ring and were placed in closed containers (24.4 ± 1 °C, 60 ± 2% RH).

The films were prepared according to mixed two-level and three-level factorial designs. The factors were the SA and the GLY concentrations, in both cases, with 2% and 3% total polymer concentration.

The prepared polymer films of different compositions can be seen in Table 1. Every second film composition contains CTZ.

### 2.3. Thickness of Films

The thickness of the films was investigated from 10 randomly selected points of all films (*n* = 10) with a screw micrometer (Mitutoyo Co. Ltd., Kawasaki, Japan), and the sensibility was 0.001 mm. The means and standard deviations were calculated from these data.

### 2.4. Tensile Strength (Hardness) of Films

**Figure 1:**
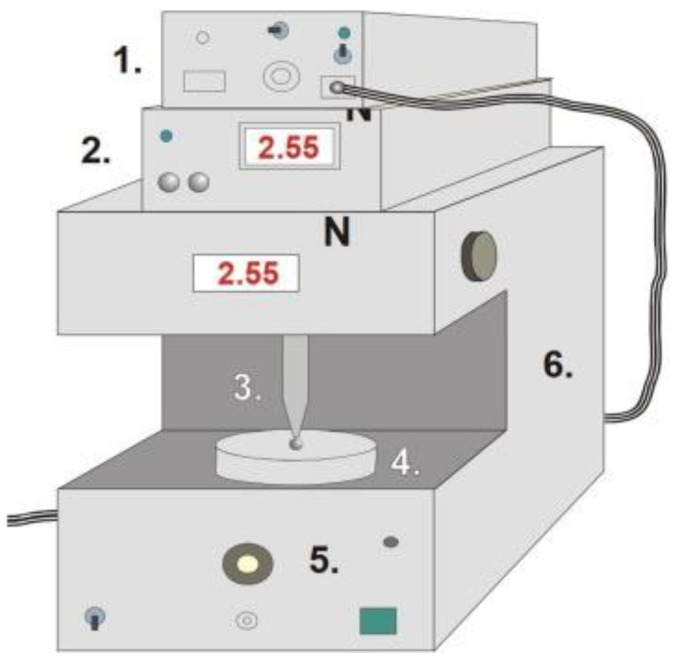
Schematic diagram of the tensile strength and mucoadhesion texture analyzer (**1**—interface, **2**—force display, **3**—rod-like or needle-like probe (jowl), **4**—sample holder, **5**—force measurement unit, and **6**—motor).

Tensile strength was investigated with a self-developed texture analyzer. The equipment and the software were developed at our institute. It has different sample holders and different probes (needle-like probe and rod-like probe). The equipment has a fixed disc (20 mm in diameter) and a moving sample holder. Depending on the investigation, a different sample holder can be used, and the force (the range was 0–200 N), moving speed (20 mm/min), and time can be registered. The needle-like probe (201 mm^2^) was used in the hardness test. The probe was moved downward at constant speed, and the sample was fixed on the bottom part of the equipment. The sampling rate was 50 Hz, and the output was 0–5 V. The probe was passed through the film. The equipment presented in Figure 1 detected the time and force during the investigation. The test was finished when the film was broken. The test was performed ten times (*n* = 10) for each combination of films. The means and standard deviations were calculated [40,41].

### 2.5. In Vitro Mucoadhesion Test

Mucoadhesion was measured with the same texture analyzer with different settings and assembling modifications. The sample holder was rod-like with a diameter of 5 mm. A double-faced adhesive tape was fixed on the surface of the sample holder, and the polymer film was fixed on the other face of the adhesive tape. A disc with a 35-mm diameter was fixed on the bottom part of the tester, and 40 µL of freshly prepared mucin dispersion (10 *w/w* %) was spread on it. The rod-like sample holder was moved downward and pressed to the mucin-covered, bottom disc with 30 ± 0.1 N for 30 s. This steady-state part can be followed in the force–time curve. After that, the sample holder was moved upwards, and the force was decreased until the sample started to separate from mucin, which can be seen as a well-defined peak in the force–time curve. The peak maximum illustrates the mucoadhesion force. The test was repeated six times (*n* = 6), and the means and standard deviations were calculated [40,41].

### 2.6. Contact Angle and Surface Free Energy (SFE) Measurement

Polymer films were placed on the microscope glass slides. One drop of distilled water and diiodomethane was used to measure the contact angle (Θ) of polymer films for 15 s, with the circle fitting method, by using a contact angle apparatus (OCA20-DataPhysics Instrument GmbH, Filderstadt, Germany). One drop of different liquids was applied, the volume of which was 10 μL and 5 µL in the cases of distilled water and diiodomethane, respectively.

The means and standard deviations (SD) were calculated from 6 identical samples of each combination of films (*n* = 6). The means and standard deviations were used to calculate the surface free energy of films. Surface free energy was calculated with Wu’s method. This method defines the amounts of the polar (*γ*^p^) and the dispersive (*γ*^d^) components of the solids. The SFE of solids can be calculated by using the following connection if the two parameters and the contact angle of the solid are known:

$$\left(1+\cos\Theta \right)\cdot \gamma =\frac{4\left(\gamma_{s}^{d}\cdot \gamma_{l}^{d}\right)}{\gamma_{s}^{d}+\gamma_{l}^{d}}+\frac{\left(\gamma_{s}^{p}\cdot \gamma_{l}^{p}\right)}{\gamma_{s}^{p}+\gamma_{l}^{p}}$$

where Θ is the contact angle of the solid–liquid surface, *γ*_l_ is the liquid surface tension, *γ*_s_ is the solid surface energy, which is the sum of their polar and dispersive components. According to Wu, the SFE of distilled water is 72.8 mN/m (polar part (*γ*^p^) = 50.2 mN/m; dispersive part (*γ*^d^) = 22.6 mN/m), while the SFE of diiodomethane is 50.8 mN/m (polar part (*γ*^p^) = 1.8 mN/m; dispersive part (*γ*^d^) = 49.0 mN/m) [42].

Knowing the polar and the dispersive parts of the polymer films, the polarity of films can be established by using the following formula:

Polarity (%) = (*γ*^p^ (mN/m)/*γ*^tot^ (mN/m)) × 100

where *γ*^tot^ (mN/m) = *γ*^p^ (mN/m) + *γ*^d^ (mN/m).

### 2.7. FTIR Spectroscopy Measurement

The Fourier-Transform Infrared Spectra of the excipients and the polymer films were investigated by using an Avatar 330 FTIR apparatus (Thermo Fisher Scientific Inc., Waltham, MA, USA) with coupled Zn/Se horizontal attenuated total reflectance (HATR) equipment. Films were laid on a clean crystal of the apparatus. The range of the wavelength was 600 to 4000 cm^−1^ during the investigation. The spectra were obtained from 128 scans, at the spectral resolution of 4 cm^−1^. CO_2_ and H_2_O were applied for correction.

### 2.8. Raman Spectroscopy Measurement

Raman spectroscopy is a promising analytical method to monitor the preparation process and to implement the Process Analytical Technology requirements. In this article, a Dispersive Raman spectrometer was used to confirm the relatively uniform distribution of API in the polymer films. Transmission Raman Spectroscopy is used for noninvasive and fast qualitative investigation of pharmaceutical dosage forms and intermediate products. In our method, the distribution of CTZ was analyzed by Raman surface mapping in CTZ-containing free buccal films.

To investigate the distribution of API, Raman spectra were acquired with a Thermo Fisher DXR Dispersive Raman (Thermo Fisher Scientific Inc., Waltham, MA, USA) equipped with a CCD camera and a diode laser operating at a wavelength of 780 nm. Raman measurements were carried out with a laser power of 24 mW at a 25-µm slit aperture size on a 2-µm spot size. The discrete spectra of the individual substances such as CTZ, HPMC, and SA, and different compositions of polymer films were collected using an exposure time of 6 s, the number of exposures was 20, and the number of background exposures was 512. Smart background was used during the whole investigation. The applied spectral range was 3200–200 cm^−1^ with cosmic ray and fluorescence corrections.

### 2.9. Dissolution Test

Polymer films of a size of 2 cm × 2 cm (containing 10 mg of CTZ) were investigated in the dissolution test. The dissolution test was made by an Erweka DT700 dissolution basket tester at a mixing speed of 100× *g* rpm. Nine hundred milliliters of phosphate buffer (pH = 6.8) was used as the dissolution medium, and its temperature was 37 °C [43]. Aliquots of 5 mL were analyzed in 5, 10, 15, 20, 30, 40, 50, 60, 90, and 120 min with Genesys 10S UV-VIS (Thermo Fisher Scientific Inc., Waltham, MA, USA) UV-spectrophotometry at *λ* = 207 nm.

### 2.10. Statistical Analysis

The collected data were analyzed with the factorial ANOVA method by Tibco Statistica v13.4.0.14 (Statsoft Inc., Tulsa, OK, USA) software. The results were evaluated by being mixed two-level and three-level factorial designs. The equations describe the relationship between the two factors (x_1_-concentration of SA and x_2_-concentration of GLY) and the four optimalization parameters (y_1_tensile strength, y_2__–_mucoadhesion force, y_3_–surface free energy, and y_4_–dissolution). The low, zero and high levels of the factors are shown in Table 2.

## 3. Results

### 3.1. Thickness and Tensile Strength of the Films

**Figure 2:**
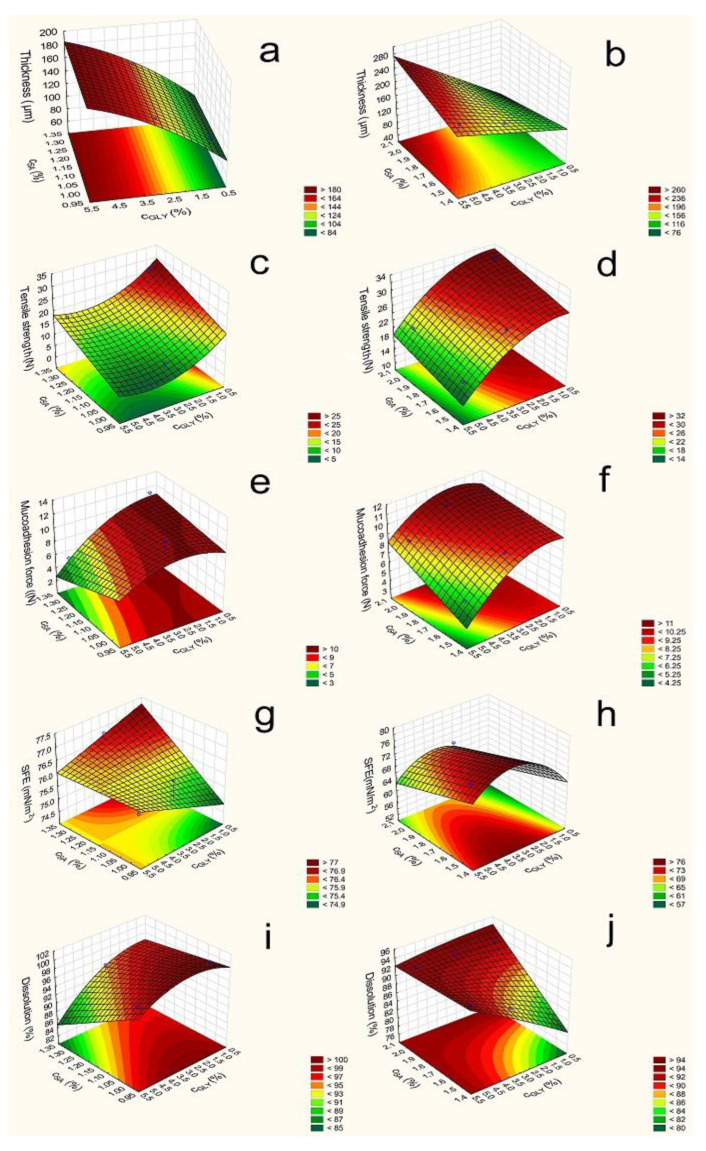
Response surface of polymer films containing CTZ (**left side**—2% polymer films; **right side**—3% polymer films; (**a**,**b**): Film thickness (2%: F (x_2_): 259.43; F (x_2_^2^): 3.27; F (x_1_x_2_): 5.12; 3%: F (x_1_): 12.48; F (x_2_): 1189.26; F (x_1_x_2_): 119.9) (**c**,**d**): Tensile strength (2%: F (x_1_): 13.61; F (x_2_): 8.0; F (x_2_^2^): 2.0; 3%: F (x_1_): 9.52; F (x_2_): 75.7; F (x_2_^2^): 4.97); (**e**,**f**): Mucoadhesion force (2%: F (x_2_): 3.03; F (x_1_x_2_): 0.7; 3%: F (x_1_): 153.54; F (x_2_): 280.33; F (x_2_^2^): 54.94; F (x_1_x_2_): 18.63); (**g**,**h**): Surface free energy (2%: F (x_2_): 0.008; F (x_2_^2^): 14.35; 3%: F (x_1_): 8.92; F(x_2_): 3.4; F (x_2_^2^): 6.82); (**i**,**j**): Dissolution (2%: F (x_1_): 15.98; F (x_2_): 9.3; F (x_2_^2^): 1.38; F(x_1_x_2_): 2.68; 3%: F (x_1_): 12.9; F (x_2_): 764.8; F (x_2_^2^): 2.68; F(x_1_x_2_): 699.21).

In Figure 2, it can be seen that the thickness (y_1_) of different compositions of film increases depends on the polymer and GLY concentration. The 3% polymer films are thicker than the 2% ones; therefore, the polymer concentration can increase the thickness of the films due to the higher amount of polymer (Equations (1)–(4)). The type of polymer does not influence the thickness notably; there is no difference between the 1:1 SA and HPMC (samples 1–6 and 13–18) and 2:1 SA and HPMC films (samples 7–12 and 19–24). It can be seen the C_SA_ (x_1_) coefficients were very low in all cases, and they were not significant. C_GLY_ (x_2_) can also influence thickness, and these were statistically significant (*p* < 0.05) in all cases. According to Gao and et al., GLY has a water retention effect, so GLY can increase the distance of bonding; therefore, the thickness of the films can be increased [30]. CTZ can also enhance the thickness, which can be explained by the fact that CTZ raises the dry matter content of the films.

y_1_ = 138.9 + 40.53∙x_2_ + 3.94∙x_2_^2^ + 5.69∙x_1_∙x_2_ (2% polymer films containing CTZ) R^2^ = 0.9218 (1)

y_1_ = 168.02 + 4.59∙x_1_ + 54.89∙x_2_ + 17.43∙x_1_∙x_2_^2^ (3% polymer films containing CTZ) R^2^ = 0.9784 (2)

y_1_ = 116.12 + 1.66∙x_1_ + 46.98∙x_2_ − 4.47∙x_1_∙x_2_ (2% polymer films without CTZ) R^2^ = 0.9461 (3)

y_1_ = 145.45 + 2.85∙x_1_ + 54.95∙x_2_ + 7.7∙x_1_∙x_2_ (3% polymer films without CTZ) R^2^ = 0.9972 (4)

The mechanical properties of the different polymer films, such as film thickness, tensile strength, mucoadhesion, are summarized numerically in Table 3.

According to Paolicelli et al., GLY can make the film soft, elastic, and flexible. In our research, it can be seen that GLY decreases the tensile strength (y_2_) of films [28]. This observation is due to the fact that GLY interacts with other components (CTZ, SA, and HPMC); typically forms H-bonds; and retains water. In addition, GLY can increase the bonding distance, so films with a high GLY concentration can break more easily.

It can be seen that the average values (14.82 and 9.55) were lower in the case of 2% polymer concentration than in the case of 3% (25.32 and 13.81). The total polymer increases the tensile strength, because it can form a cohesive, strong, stable structure in the films due to the bonding of polymer chains. On the other hand, our results show that a higher amount of SA (x_1_) (samples 7–12 and 19–24) can result in a stronger structure compared to an equal amount of the polymer. This is shown by the value of the coefficients x_1_ shown in (Equations (5)–(8)), which is positive in all cases and statistically significant in two cases (Equations (7) and (8)). It is possible that SA can create stronger and a larger number of bonds with CTZ than HPMC can with CTZ and itself. Another finding is that 3% polymer films have larger strengths than 2% polymer films. 

Finally, it can be concluded that 2% polymer films with lower c_SA_ (samples 1–6) have low tensile strengths (less than 10 N), and these films are very breakable. This property is not appropriate from the aspect of application. The other film compositions have high tensile strength and can be used properly for buccal application, because they do not break from the force of the finger.

The different optimalization parameters were determined by a statistical analysis. In the first step, the tensile strength was investigated. The data and Figure 2 reveal that, in the 2% polymer films, SA and GLY have a significant effect. The SA concentration can increase the tensile strength, but GLY has a reverse effect on the tensile strength; it can decrease the tensile strength of the films. In the 3% polymer films, we can see a similar effect, but the effect of GLY is not significant. Equations (Equations (5)–(8)) can be seen below in the case of the different compositions. The significant parameters are marked with red letters.

Tensile strength:

y_2_ = 14.82 + 4.79∙x_1_ − 4.50∙x_2_ − 1.95∙x_2_^2^ (2% polymer films containing CTZ)(5)

R^2^ = 0.9218 y_2_ = 25.32 + 1.72∙x_1_ − 5.93∙x_2_ + 1.32∙x_2_^2^ (3% polymer films containing CTZ) R^2^ = 0.9784 (6)

y_2_ = 9.55 + 3.81∙x_1_ − 2.29∙x_2_ − 2.24∙x_1_∙x_2_ (2% polymer films without CTZ) R^2^ = 0.9955 (7)

y_2_ = 13.81 + 5.66∙x_1_ − 1.24∙x_2_ + 2.70∙x_2_^2^ + 0.73∙x_1_∙x_2_ (3% polymer films without CTZ) R^2^ = 0.9979 (8)

### 3.2. In Vitro Mucoadhesion Test

The results of the mucoadhesion of the prepared films are presented in Figure 2. The CTZ-free films had high mucoadhesion force (y_3_) values, and the average values were 18.96 (Equation (11)) and 23.82 (Equation (12)). The CTZ-containing samples had significantly lower mucoadhesion force (average value: 8.41 and 9.1 (Equations (9) and (10)), so CTZ decreases the mucoadhesion of polymer films, possibly due to the interaction between the carboxylic groups of SA, HPMC, and CTZ molecules; thus, fewer groups are able to bind to the mucin. In the case of these samples, mucoadhesion is moderate, but they can be used for the buccal drug delivery system, because this lower force is enough for buccal mucoadhesion. The increasing total amount of polymers increased the mucoadhesion due to the higher number of free binding groups in the system, which can bind to the mucin of the buccal mucous.

GLY (x_2_) can influence the mucoadhesion force. The increasing amount of GLY decreased the mucoadhesion force, probably because of the hydrogen bond, which can be formed between GLY and the film-forming polymers and, also, due to the fact that no binding is formed with the chains of mucin because of the lower number of free chains in the polymer.

During the statistical analysis, the mucoadhesion force of the films of all compositions (CTZ-free and CTZ containing) was found to be decreased by GLY (x_2_), and in the case of films prepared with 3% polymer concentrations, it is statistically significant. The c_SA_ (x_1_) slightly decreased or did not affect it, because SA has a moderate mucoadhesion force, while HPMC has a higher one, so a greater HPMC concentration can cause higher mucoadhesion in the films, but this effect was not statistically significant.

Mucoadhesion force:

y_3_ = 8.41 − 2.09∙x_2_ − 1.01∙x_1_∙x_2_ (2% polymer films containing CTZ) R^2^ = 0.6133 (9)

y_3_ = 9.10 + 0.92∙x_1_ − 1.53∙x_2_ + 0.58∙x_2_^2^ + 0.39∙x_1_∙x_2_ (3% polymer films containing CTZ)(10)

R^2^ = 0.9981 y_3_ = 18.96 − 1.21∙x_1_ − 4.47∙x_2_ + 0.44∙x_1_∙x_2_ (2% polymer films without CTZ) R^2^ = 0.8408 (11)

y_3_ = 23.82 − 1.53∙x_1_ − 2.96∙x_2_ (3% polymer films without CTZ)
R^2^ = 0.9120 (12)

### 3.3. Contact Angle and Surface Free Energy (SFE) Measurement

The SFEs (y_4_) of the prepared films are shown in Table 4. This measurement is very important from the aspect of applying this pharmaceutical form, because the saliva has to spread on the surface of the films extensively in order for mucoadhesion to develop. The CTZ-free films exhibited medium SFE 51.99 and 57.81 average values (Equations (15) and (16)), while the CTZ-containing films have significantly higher SFE, the average value was 69 and 76.11 (Equations (13) and (14)), so CTZ can increase the SFE of films, particularly the polar part of SFE. CTZ films also demonstrated significantly higher polarity, which may be caused by the carboxylic group of the CTZ molecule. From these results, it seems to be an extended conformer of CTZ can be found in the prepared films [34]. Contact angle showed lower values in the CTZ containing films. The SFE of CTZ-free films changed particularly as a function of increasing GLY concentration (x_2_) and only slightly as a function of the SA concentration (x_1_). This observation was not true for the CTZ films, because in those films, the SFEs had roughly constant values independently of the concentration of GLY and polymer concentration (Figure 2).

During the next step of the statistical analysis, SFE was determined in the different compositions of films. In the films, containing CTZ, SA, and GLY can increase SFE in the 2% polymer films, while in the 3% CTZ-free films SA decreases SFE. See Equations (13)–(16) below.

Surface free energy:

y_4_ = 76.11 + 0.46∙x_1_ + 0.375∙x_2_^2^ − 0.43∙x_1_∙x_2_ (2% polymer films containing CTZ) R^2^ = 0.9638 (13)

y_4_ = 69.00 − 3.04∙x_1_ + 2.30∙x_2_ + 2.82∙x_2_^2^ (14)

(3% polymer films containing CTZ) R^2^ = 0.9054 y_4_ = 57.81 + 3.19∙x_1_ + 5.67∙x_2_ + 0.84∙x_2_^2^ (2% polymer films without CTZ) R^2^ = 0.7854 (15)

y_4_ = 51.99 − 2.19∙x_1_ + 2.04∙x_1_∙x_2_ (3% polymer films without CTZ) R^2^ = 0.7237 (16)

### 3.4. FTIR Measurement

**Figure 3:**
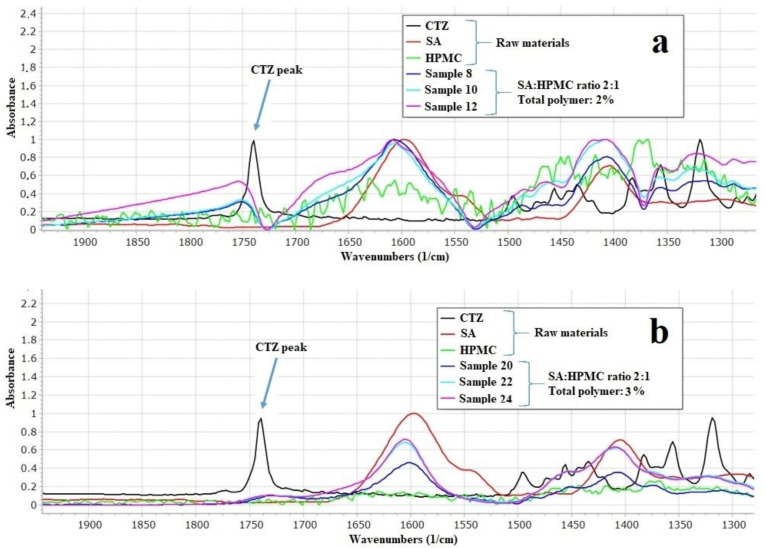
Individual FTIR spectra of SA, HPMC, CTZ, and prepared films (**a**): 2% polymer films and (**b**): 3% polymer films.

The prepared films were investigated with FTIR spectroscopy to determine the interactions between the components of the films. According to Paczkowsa et al., the carboxyl group of CTZ can be explored at 1739 cm^−1^ in FTIR spectra (Figure 3) [44]. In the spectra of CTZ, this peak can be separated sharply. However, in the films, this peak is shifted and disappears, depending on the concentration of the polymer. In the 2% polymer films, the CTZ peak is shifted towards the larger wavenumber, but the intensity of the peak is smaller than in the case of the raw material. As for 3% polymer films, the peak disappears completely, which proves that interactions can develop between CTZ and the polymers in the films. The carboxyl group of CTZ can be found at 1739 cm^−1^, so it can be asserted that hydrogen bondings are created between SA, HPMC, and the carboxylic group of CTZ. Due to the small polymer concentration, the films have fewer OH groups of SA and HPMC, which can create hydrogen bonding, so, in this case, the peak only just shifted. However, the 3% polymer films have more OH groups, thus more hydrogen bonds can be created, which can cause a more remarkable structure change, so the peak can disappear completely.

Indeed, there are important bands in the higher wavenumber region. A spectral peak is manifested at 2384 cm^−1^; this is the stretching vibration of the N-H group of CTZ [45]. In both 2% and 3% concentration polymer films, this peak disappears regardless of the GLY concentration. This observation can suggest that, in the films interaction develop between the N-H-group of CTZ and the other components of films. A peak of SA can be detected at 2937 cm^−1^ [46]. This peak is assigned to the C-H stretching vibration. In the polymer films, this peak is shifted to a larger wavenumber and merges with the peak of GLY. The band at 3300 cm^−1^ shows the C-O stretching vibration. The intensity of this peak can increase as a function of GLY concentration and is shifted to a smaller wavenumber. With these findings, we believe we have sufficiently demonstrated the interactions that can occur in the films.

As it can be seen, the dissolution of the API is faster from the 2% polymer films, which contain the two different polymer materials in equal amounts than from those films that contain SA and HPMC in the ratio of 2:1 (it can be found in Figure 3 in Section 3.4, in the dissolution test part of the article). This observation can be explained by the interaction between SA and GLY and other components at 2937^−1^ cm^−1^. The dependence of the GLY concentration of the drug release is related to the interaction at 3300 cm^−1^. The high GLY concentration can result in a stronger interaction between the components of the films, as can be seen in the region from 3000 cm^−1^ to 2800 cm^−1^ and in the region 1750 cm^−1^ and 1550 cm^−1^. The results of Raman spectroscopy also confirm these interactions.

### 3.5. Raman Spectroscopy Measurement

**Figure 4:**
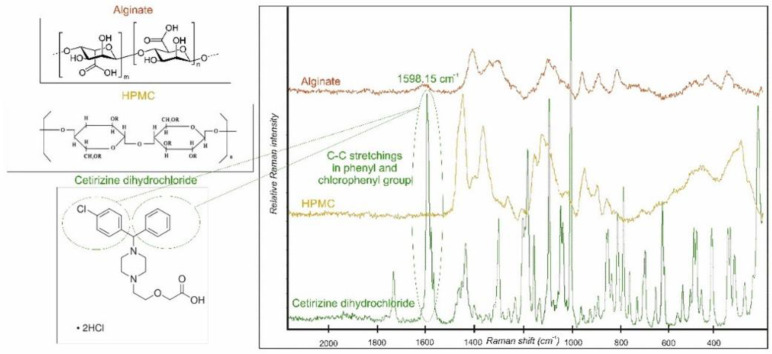
Individual Raman spectra of SA, HPMC, and CTZ.

The individual Raman spectra of SA, HPMC, and CTZ are shown in Figure 4. Based on the comparison of the spectra of the components, a CTZ peak was chosen at 1598.15 cm^−1^. It belongs to the C-C stretchings in the phenyl and chlorophenyl groups in the chemical structure of CTZ [44]. This distinct peak was chosen for the chemical mapping profile, because it characterizes the API individually.

**Figure 5:**
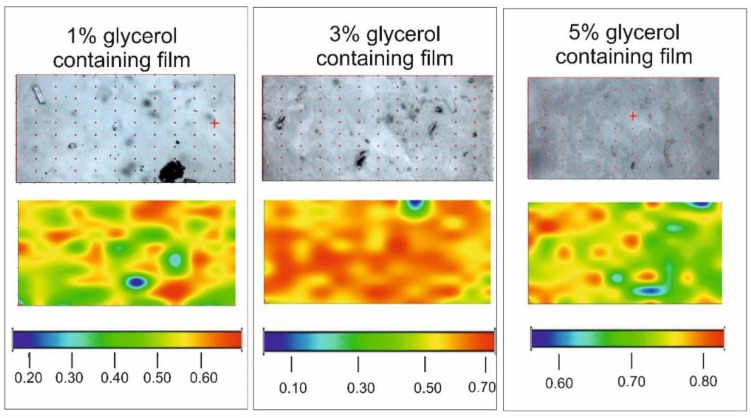
Chemical mapping of films with different GLY contents.

Chemical maps are presented in Figure 5. The CTZ distributions of samples with different GLY content are homogenous. The warm (red and orange) colors mean a greater API content in the samples. In the film with 1% GLY content the API can be in crystalline agglomerates (yellow, orange spots) in the preparation. In the two other cases, especially in the case of the sample containing 3% GLY, the color distribution of the maps is very smooth. It suggests a possible molecular disperse distribution of the API in the film. It can be concluded that the water-soluble API is in a dissolved form in the films because the larger GLY concentration also means larger water content because of the moisturizing property of GLY. This property of the films can be disadvantageous, because it can cause the physical instability of the samples. Besides, the larger GLY concentration can be harmful during the mucoadhesion of buccal films because GLY reduces the amount of mucoadhesive polymer chains, which are able to evolve binding.

### 3.6. Dissolution Test

**Figure 6:**
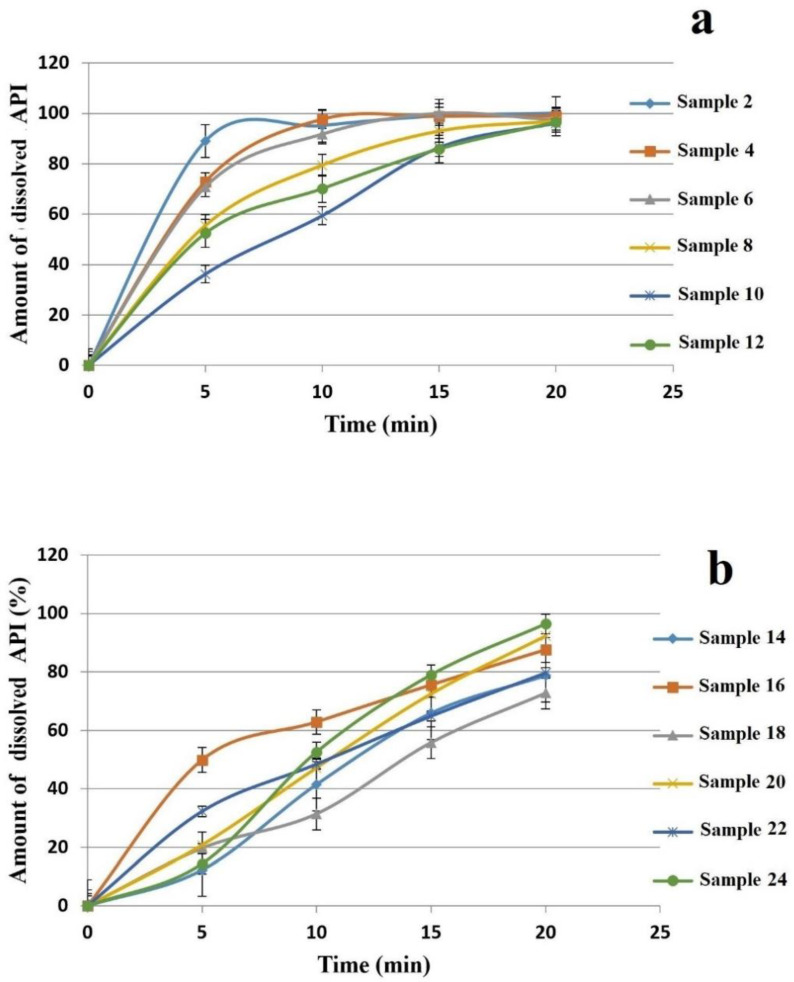
Dissolution curves of prepared polymer films in the first 20 min (**a**): 2% polymer films, and (**b**): 3% polymer films).

The entire amount of the API can be dissolved from the various films within 120 min. In the first 20 min, almost 100% of the API can be dissolved from the films with 2% polymer concentration (Figure 6). Centkowska et al. found that the thickness of the HPMC films can influence the release of API from the film [47]. CTZ can dissolve from the thinner polymer layer faster than from thicker films, and on the other hand, in the case of thinner films, HPMC is able to dissolve more easily. Films with a lower SA concentration have faster API dissolution, which can be slowed down by GLY. When the SA and GLY concentrations were increased, dissolution became slower in the case of 2% polymer concentration.

The dissolution curve of 3% polymer films is shown in Figure 6, which reveals a connection between the SA concentration and dissolution rates. In about 120 min, the entire amount of the API can be dissolved completely, but in the first 20 min, the API cannot be released fully; 65% to 96% of the API was dissolved from the different composition films. It is noteworthy in the figure that, at the beginning of the test (first 5 min), the API was dissolved faster from the films with equal polymer concentration. The reason for this is that in the case of 3% polymer concentration a stable, cohesive structure is formed between the chains of the polymers, GLY and API, as visible from the thickness and hardness tests; therefore, GLY cannot affect the dissolution significantly. Compared to 2% films, the effect of GLY is less than expected in 3% films.

When the dissolved drug was investigated after 20 min with a statistical analysis, it can be seen that, in the 2% polymer films c_SA_ (x_1_) can decrease it, while in the 3% films c_SA,_ can enhance it. c_GLY_ (x_2_) can raise the dissolution rate when high SA concentration is applied (Figure 2). Equations (17) and (18) can be observed below.

Dissolution:

y_5_ = 95.66 − 3.1∙x_1_ − 2.89∙x_2_ + 0.97∙x_2_^2^ − 1.55∙x_1_∙x_2_ (2% polymer films containing CTZ) R^2^ = 0.9671 (17)

y_5_ = 89.85 + 2.54∙x_1_ + 2.40∙x_2_ + 0.12∙x_2_^2^ − 2.29∙x_1_∙x_2_ (3% polymer films containing CTZ) R^2^ = 0.9996 (18)

## 4. Conclusions

In this present study we successfully formulated mucoadhesive polymer films, which contained CTZ as API. The physical properties of the films, which are of great importance in the formulation of polymer films, were investigated. We can conclude that the physical properties of the polymer films, such as thickness and tensile strength, change considerably depending on the composition of films. These are critical parameters of the formulation because buccal films have to be elastic, flexible, and able to exert satisfactory mucoadhesive force at the same time. The _GLY_ was a statistically significant factor in the case of film thickness and tensile strength for 3% total polymer film but the coefficient also had a high value in the case of 2% total polymer. In the mucoadhesion test, c_GLY_ was found to be a significant factor with the inverse relationship. It can decrease mucoadhesion force; therefore, only 1% GLY concentration is recommended.

CZT-containing films had lower contact angle values, the fluid could spread on the surface of the film. It proves that CTZ-containing films have a hydrophilic surface. CTZ can also increase SFEs, because it has hydrophilic molecule groups, and it can increase the hydrophilicity of the polymer film system. In these cases, no statistically significant factor was found.

During the interaction study, interactions could be observed between the polymer components and the CTZ molecules, which depended on the polymer concentration. In the 3% polymer films, the development of hydrogen bonds is remarkable.

The distribution of CTZ is homogeneous in the films, so the preparation of the films can be considered appropriate. GLY can influence the form of CTZ in the films. In the films with 1% GLY concentration CTZ is crystalline, while in the films with 3% and 5% GLY concentration the API is molecular disperse.

In 20 min, a higher amount of API can dissolve from the thicker and lower polymer concentration polymer film samples, because they are not able to form a stable, strong structure, so the dissolution of the API is faster and easier. In the film samples with a higher polymer concentration, the polymer molecules and GLY and CTZ can create strong bonds, so CTZ can be dissolved more slowly. GLY can decrease the dissolution rate of CTZ.

As a conclusion, samples with 1% of GLY were found to be appropriate for the application on the buccal mucosa. Sample 8 was found as the optimal composition.

We are planning to continue to investigate the films with in vitro permeation tests on buccal cell lines, with ex vivo permeation tests across porcine buccal mucosa in the future. We would like to publish these results in another article together with the results of the accelerated stability test.

## Figures and Tables

**Table 1:** Composition of the prepared SA-based films.

Total Polymer (%)	Samples	SA (*w/w* %)	HPMC (*w/w* %)	GLY (*w/w* %)	CTZ (10 mg)
2	1	1	1	1	−
	2	1	1	1	+
	3	1	1	3	−
	4	1	1	3	+
	5	1	1	5	−
	6	1	1	5	+
	7	1.33	0.66	1	−
	8	1.33	0.66	1	+
	9	1.33	0.66	3	−
	10	1.33	0.66	3	+
	11	1.33	0.66	5	−
	12	1.33	0.66	5	+
3	13	1.5	1.5	1	−
	14	1.5	1.5	1	+
	15	1.5	1.5	3	−
	16	1.5	1.5	3	+
	17	1.5	1.5	5	−
	18	1.5	1.5	5	+
	19	2	1	1	−
	20	2	1	1	+
	21	2	1	3	−
	22	2	1	3	+
	23	2	1	5	−
	24	2	1	5	+

**Table 2:** The values of factors for mixed two-level and three-level factorial designs.

Total Polymer Concentration (%)	Factors	Low Level	Zero Level	High Level
2%	Concentration of SA (x_1_)	1	-	1.33
	Concentration of GLY (x_2_)	1	3	5
3%	Concentration of SA (x_1_)	1.5	-	2
	Concentration of GLY (*x*_2_)	1	3	5

**Table 3:** Mechanical properties of the various polymer films.

Samples	Thickness (µm)	Tensile Strength (N)	Mucoadhesion (N)
1	56.25 ± 8.54	5.61 ± 0.01	16.94 ± 1.20
2	104.75 ± 7.27	13.89 ± 0.92	8.56 ± 1.52
3	128.00 + 7.7	6.07 ± 1.07	15.70 ± 1.32
4	142.32 ± 8.52	9.90 ± 1.19	11.94 ± 1.31
5	159.14 ± 6.32	5.50 ± 1.95	7.13 ± 2.04
6	174.43 ± 6.02	6.33 ± 0.75	6.38 ± 1.59
7	81.50 ± 6.66	18.15 ± 1.45	16.02 ± 2.20
8	86.74 ± 6.34	27.37 ± 0.28	11.57 ± 1.45
9	105.31 ± 8.18	12.85 ± 2.37	8.54 ± 1.10
10	146.00 ± 6.06	14.56 ± 2.18	6.63 ± 1.25
11	166.52 ± 3.87	9.10 ± 1.91	7.97 ± 2.23
12	179.19 ± 4.99	16.92 ± 1.46	5.37 ± 1.64
13	93.53 ± 6.35	8.53 ± 0.57	22.12 ± 2.11
14	125.29 ± 5.56	28.06 ± 3.76	9.66 ± 0.70
15	146.22 ± 5.91	11.39 ± 0.91	17.59 ± 0.63
16	164.79 ± 5.88	26.47 ± 1.42	9.07 ± 1.95
17	188.03 ± 2.71	4.58 ± 0.37	15.64 ± 1.41
18	200.22 ± 8.14	16.28 ± 2.92	5.83 ± 2.11
19	87.23 ± 1.26	17.96 ± 1.52	17.32 ± 2.74
20	102.00 ± 2.71	32.68 ± 3.03	10.82 ± 1.99
21	145.12 ± 3.46	23.47 ± 2.49	16.87 ± 1.51
22	169.20 ± 2.49	27.68 ± 3.65	10,7 ± 2.33
23	212.55 ± 9.98	16.96 ± 1.95	11.95 ± 0.30
24	246.65 ± 5.51	20.75 ± 0.22	8.56 ± 1.87

**Table 4:** Surface free energy (***γ*^tot^**), dispersive (*γ*^d^), and polar part (*γ*^p^) of SFE and the polarity (%) of prepared films.

Samples	*γ*^tot^ (mN/m)	*γ*^d^ (mN/m)	*γ*^p^ (mN/m)	Polarity (%)
1	49.59	32.54	17.05	34.38
2	74.88	38.85	36.03	48.12
3	51.65	36.34	15.31	29.64
4	76.33	37.44	38.89	50.95
5	62.60	36.35	26.25	41.93
6	75.72	35.60	40.12	52.98
7	53.56	32.28	21.29	39.75
8	76.86	39.17	37.69	49.04
9	66.21	31.85	34.36	51.90
10	76.89	40.57	36.32	47.24
11	63.23	28.15	35.08	55.48
12	75.98	38.26	37.73	49.66
13	54.32	33.12	21.20	39.03
14	68.61	38.80	29.87	43.54
15	57.36	36.21	21.15	36.87
16	73.78	35.14	38.65	52.39
17	50.84	33.59	17.25	33.93
18	73.72	36.07	37.65	51.07
19	47.99	33.56	14.43	30.07
20	61.05	35.11	25.93	42.47
21	48.72	31.61	17.11	35.12
22	71.73	37.12	34.61	48.25
23	52.68	36.01	16.67	31.64
24	65.12	38.40	26.72	41.03

## Data Availability

Data sharing not applicable.

## Abbreviations

Sodium alginate, SA; glycerol, GLY; hydroxypropyl methylcellulose, HPMC; cetirizine dihydrochloride, CTZ; surface free energy, active pharmaceutical ingredient, API; surface free energy, SFE; Fourier-Transform Infrared Spectra, FTIR; standard deviation, SD.
